# Bufalin Induces Lung Cancer Cell Apoptosis via the Inhibition of PI3K/Akt Pathway

**DOI:** 10.3390/ijms13022025

**Published:** 2012-02-14

**Authors:** Zhitu Zhu, Hongzhi Sun, Guangyou Ma, Zhenghua Wang, Enze Li, Yangyang Liu, Yunpeng Liu

**Affiliations:** 1 Department of Oncology, the First Affiliated Hospital of Liaoning Medical University, Jinzhou, Liaoning 121001, China; E-Mails: cmushz@163.com (H.S.); heroiheart@163.com (G.M.); wangzhenghua1977@163.com (Z.W.); dxjlez@163.com (E.L.); zhmeiyan211@163.com (Y.L.); 2 Department of Oncology, the First Affiliated Hospital of Chinese Medical University, Shenyang, Liaoning 120000, China; E-Mail: cmuliuyunpeng@yahoo.cn

**Keywords:** lung cancer, Apoptosis, bufalin, PI3K/Akt, A549 cells

## Abstract

Bufalin is a class of toxic steroids which could induce the differentiation and apoptosis of leukemia cells, and induce the apoptosis of gastric, colon and breast cancer cells. However, the anti-tumor effects of bufalin have not been demonstrated in lung cancer. In this study we used A549 human lung adenocarcinoma epithelial cell line as the experimental model to evaluate the potential of bufalin in lung cancer chemotherapy. A549 cells were treated with bufalin, then the proliferation was detected by MTT assay and apoptosis was detected by flow cytometry analysis and Giemsa staining. In addition, A549 cells were treated by Akt inhibitor LY294002 in combination with bufalin and the activation of Akt and Caspase-3 as well as the expression levels of Bax, Bcl-2 and livin were examined by Western blot analysis. The results showed that Bufalin inhibited the proliferation of A549 cells and induced the apoptosis of A549 cells in a dose and time dependent manner. Mechanistically, we found that bufalin inhibited the activation of Akt. Moreover, bufalin synergized with Akt inhibitor to induce the apoptosis of A549 cells and this was associated with the upregulation of Bax expression, the downregulation of Bcl-2 and livin expression, and the activation of Caspase-3. In conclusion, our findings demonstrate that bufalin induces lung cancer cell apoptosis via the inhibition of PI3K/Akt pathway and suggest that bufalin is a potential regimen for combined chemotherapy to overcome the resistance of lung cancer cells to chemotherapeutics induced apoptosis.

## 1. Introduction

Lung cancer is one of the most common malignant tumors. Non-small cell lung cancer (NSCLC) accounts for nearly 85% of all lung cancer cases and the majority of them are diagnosed at advanced stages. The 5-year survival rate of NSCLC patients has only been increased to 15% despite recent development in chemotherapy [1]. Current chemotherapy protocols for NSCLC mainly include the combination of classic drugs, such as cisplatin or carboplatin, with new drugs, such as gemcitabine, vinorelbine, paclitaxel, or docetaxel [2,3]. However, these drugs for NSCLC are known to induce chemoresistance and have a range of side effects such as hair loss, nerve damage, hearing loss, kidney damage, or allergic reactions. Therefore, it is urgent to develop novel chemotherapies that are highly effective and less toxic.

Bufalin is a class of toxic steroids purified from Chinese traditional medicine chan'su. Recent studies have shown that bufalin exhibits anti-tumor effects [4,5]. Bufalin could induce the differentiation and apoptosis of leukemia K562 and HL-60 cells, and induce the apoptosis of gastric cancer cells, colon cancer cells and breast cancer cells [6,7]. However, the anti-tumor effects of bufalin have not been demonstrated in lung cancer.

Resistance to apoptosis is an important hallmark of tumor cells [8]. Apoptosis of tumor cells is known to be regulated by a variety of signaling pathways such as PI3k/Akt pathway. Akt has been shown to regulate apoptosis related proteins such as Bcl-2, Bax and caspase-3 and is crucially involved in anticancer drug induced apoptosis of cancer cells [9–12].

Based on previous reports, we hypothesized that bufalin could induce the apoptosis of lung cancer cells via the regulation of PI3K/Akt pathway. In this study we used A549 human lung adenocarcinoma epithelial cell line as the experimental model to test our hypothesis. Our results showed that bufalin could modulate the expression of apoptosis related proteins and induce the apoptosis of A549 cells, suggesting its potential application in lung cancer chemotherapy.

## 2. Results and Discussion

### 2.1. Bufalin Inhibits the Proliferation and Induces the Apoptosis of A549 Cells

**Figure 1:**
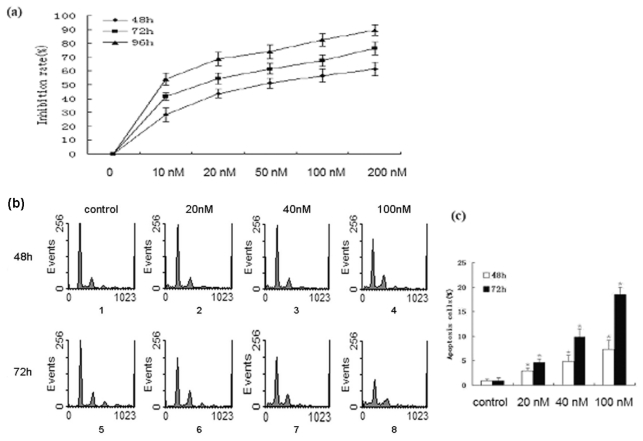
Bufalin inhibits the proliferation and induces the apoptosis of A549 cells. (**a**) A549 cells were treated with 10 nmol/L, 20 nmol/L, 50 nmol/L, 100 nmol/L or 200 nmol/L bufalin for 48 h, 72 h, or 96 h. The cell viability was examined by MTT assay. Data were derived from three independent experiments. (**b**) A549 cells were treated with 20 nmol/L, 40 nmol/L, or 100 nmol/L bufalin for 48 h or 72 h. The apoptosis was examined by PI staining and flow cytometry analysis. (**c**) Quantification of apoptosis of A549 cells shown in (**b**). Data were derived from three independent experiments.* *P* < 0.01 *vs.* control.

To investigate the effects of bufalin on A549 cells, we tested the effect of various doses of bufalin on the viability of A549 cells using MTT assay. The cells were treated for 48 h, 72 h, or 96 h. as shown in Figure 1a, cell growth was inhibited by bufalin in a dose and time dependent manner. IC_50_ was calculated as 56.14 ± 6.72, 15.57 ± 4.28, and 7.39 ± 4.16 nmol/L for cells treated at 48 h, 72 h and 96 h, respectively.

We speculated that the reduced viability of A549 cells may be caused by the induction of apoptosis, thus we carried out PI/flow cytometry analysis to examine the apoptosis. A549 cells were treated with 20 nmol/L, 40 nmol/L, or 100 nmol/L bufalin for 48 h or 72 h. Flow cytometry analysis showed that bufalin induced the apoptosis of A549 cells in a dose and time dependent manner (Figure 1b). Qualification of the apoptosis rate showed that the differences in apoptosis rate between control cells and cells treated with bufalin were significant (*p* < 0.01, Figure 1c).

**Figure 2:**
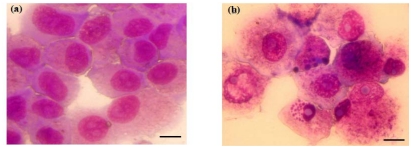
Apoptotic morphology of A549 cells treated with bufalin. A549 cells were treated with vehicle (**a**) or 100 nmol/L bufalin (**b**) for 48 h and stained by Giemsa staining. cytoplasmic shrinkage, nuclear condensation and the formation of apoptotic bodies were observed in (**b**). Shown are representative images of three independent experiments. Scale bar: 40 μm.

To confirm that bufalin induces the morphology of apoptosis in A549 cells, we treated A549 cells with 100 nmol/L bufalin for 48 h and then performed Giemsa staining. The apoptotic morphology was obviously observed in bufalin treated cells under a microscope at 200× magnification, including cytoplasmic shrinkage, nuclear condensation and the formation of apoptotic bodies (Figure 2). However, no apoptotic morphology was observed in control treated cells.

### 2.2. Bufalin Regulates the Expression of Apoptosis Related Proteins in A549 Cells

**Figure 3:**
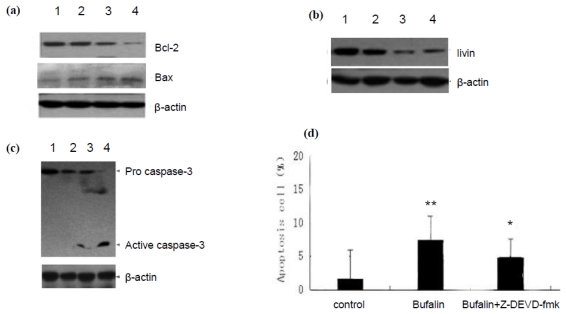
Bufalin regulates the expression of apoptosis related proteins in A549 cells. (**a**) Western blot analysis of Bcl-2 ad Bax protein level in A549 cells treated with 100 nmol/L bufalin for different time. (**b**) Western blot analysis of livin protein level in A549 cells treated with 100 nmol/L bufalin for different time. (**c**) Western blot analysis of the levels of pro-caspase-3 and activated Caspase-3 in A549 cells treated with 100 nmol/L bufalin for different time: 1. 0 h; 2. 12 h; 3. 24 h; 4. 48 h. β-actin served as loading control. Shown are representative blots from three independent experiments with similar results. (**d**). A549 cells were treated with vehicle, 100 nmol/L bufalin alone or plus Z-DEVD-fmk and the apoptosis was examined by PI staining and flow cytometry analysis. Data were derived from three independent experiments.* *P* <0.01 *vs.* bufalin alone; * *P* <0.01 *vs.* control.

Next we explored the molecular mechanism by which bufalin induces the apoptosis of A549 cells. A549 cells were treated with 100 nmol/L bufalin for 12 h, 24 h or 48 h and subjected to Western blot analysis. The results showed that bufalin increased Bax expression while decreased Bcl-2 expression at protein level in a time dependent manner (Figure 3a). Compared to control, the Bax/Bcl-2 ratio was 1.27, 1.73, and 2.68 at 12 h, 24 h and 48 h, respectively (*P* < 0.05, *P* < 0.01, *P* < 0.01, respectively). In addition, we found that livin expression level was reduced to 61.29 ± 7.76%, 29.03 ± 4.63% and 22.17 ± 6.02% of the control at 12 h, 24 h and 48 h, respectively (*P* < 0.01, Figure 3b). These results were expected because Bax is apoptosis promoting protein while Bcl-2 and livin are anti-apoptosis proteins.

Furthermore, we examined the activation of apoptosis effector Caspase-3 by detecting the cleaved form of Caspase-3. With the prolongation of bufalin treatment, the amount of cleaved form of Caspase-3 (17 KD) increased (Figure 3c). To confirm that bufalin regulates the activation of Caspase-3, we employed Z-DEVD-fmk, a specific inhibitor of Caspase-3, and found that it could inhibit bufalin induced apoptosis of A549 cells (Figure 3d). Taken together, these results suggest that bufalin regulates the expression of apoptosis related proteins and the activation of Caspase-3 to induce the apoptosis of A549 cells.

### 2.3. Bufalin Modulates the Activation of PI3K/Akt Pathway in A549 Cells

**Figure 4:**
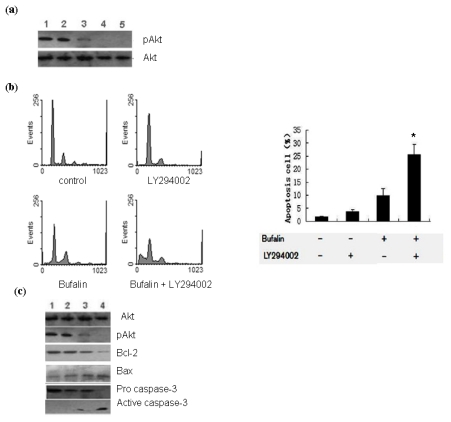
Bufalin modulates the activation of PI3K/Akt pathway in A549 cells. (**a**) Western blot analysis of the level of p-Akt in differently treated A549 cells. Total Akt level served as loading control. Shown are representative blots from three independent experiments with similar results: 1. 0 h; 2. 100 nmol/L bufalin for 24 h; 3. 100 nmol/L bufalin for 48 h; 4. 25 μmol/L LY294002 for 2 h plus 100 nmol/L bufalin for 24 h; 5. 25 μmol/L LY294002 for 2 h plus 100 nmol/L bufalin for 48 h. (**b**) A549 cells were pretreated with 25 μmol/L LY294002 for 2 h followed by treatment with 100 nmol/L bufalin for 48 h as indicated and the apoptosis was examined by PI staining and flow cytometry analysis. Data were derived from three independent experiments. * *P* < 0.01 *vs.* left three groups of cells. (**c**) Western blot analysis of the levels of Bcl-2, Bax, livin and activated caspase-3 in differently treated A549 cells. Total Akt served as loading control. Shown are representative blots from three independent experiments with similar results: 1. 0 h; 2. 100 nmol/L bufalin for 24 h; 3. 25 μmol/L LY294002 for 2 h; 4. 25 μmol/L LY294002 for 2 h plus 100 nmol/L bufalin for 24 h.

To test our hypothesis that bufalin could induce the apoptosis of lung cancer cells via the regulation of PI3K/Akt pathway, first we detected the activation of Akt in A549 cells treated with 100 nmol/L bufalin. Western blot analysis showed that the level of p-Akt was reduced in bufalin treated cells compared to control cells, although the total level of Akt remained the same (Figure 4a). These results indicate that bufalin inhibits the activation of Akt in A549 cells. To provide further evidence, we employed specific Akt inhibitor LY2942002. Pretreatment with 25 μmol/L LY294002 for 2 h could significantly augment the inhibitory effects of bufalin on Akt activation (Figure 4a).

In addition, we found that pretreatment with LY294002 (25 μmol/L) could synergize with bufalin treatment (100 nmol/L) to induce the apoptosis of A549 cells as assessed by flow cytometry analysis (Figure 4b). Moreover, we examined the synergistic effects of Akt inhibitor and bufalin on the expression of apoptosis related proteins and the activation of Caspase-3. Western blot analysis demonstrated that LY294002 pretreatment (25 μmol/L for 2 h) could synergize with bufalin treatment (100 nmol/L for 48 h) to upregulate Bax expression, downregulate Bcl-2 and livin expression, and promote the activation of Caspase-3 (Figure 4c). These results suggest that bufalin could inhibit the activation of PI3k/Akt pathway to induce the apoptosis of A549 cells.

## 3. Materials and Methods

### 3.1. Reagents and Antibodies

Bufalin was purchased from Sigma-Aldrich (St. Louis, MO, USA) and was dissolved in ethyl alcohol to make 0.01 mol/L stock solution, which was kept at −20 °C and diluted in phosphate buffer saline (PBS) when used. LY294002 was purchased from Sigma-Aldrich (St. Louis, MO, USA) and was dissolved in DMSO to make 0.01 mol/L stock solution, which was kept at 4 °C. Mouse antibodies specific for Caspase-3, Bcl-2, livin, p-Akt, Akt, and β-actin were purchased from Santa Cruz Biotechnology (Santa Cruz, CA, USA). Horseradish peroxidase-conjugated goat anti-mouse secondary antibodies were obtained from Santa Cruz Biotechnology.

### 3.2. Cell Culture

A549 human lung adenocarcinoma epithelial cell line was obtained from Cell Bank of Shanghai Institute of Biochemistry & Cell Biology, Chinese Academy of Sciences (Shanghai, China), where they were tested and authenticated based on cross species checks, DNA authentication and quarantine. A549 cells were cultured at 37 °C with 5% CO_2_ in an air atmosphere in F12K medium (Sigma), supplemented with 10% fetal calf serum (FCS) and 1% penicillin/streptomycin. A549 cells at exponential growth stage were employed in all of the experiments.

### 3.3. MTT Assay

MTT assay was employed to examine the effects of bufalin on the proliferation of A549 cells. Briefly, the cells were seeded in 96-well plates at 5 × 10^3^ cells/well in 180 μL medium and cultured for 12 h to allow attachment. Then the cells in the wells were treated with fresh medium containing different concentrations of bufalin diluted from stock solution. PBS was added to the wells as negative control. The cells were cultured for 48 h, 72 h, or 96 h. Four hours before the end of culture, MTT solution (5 mg/mL in 20 μL PBS) was added to each well and incubated for 4 h at 37 °C. The growth medium was then removed and replaced with DMSO (200 μL/well), and incubated for 10 min. A MR7000 microplate reader (Dynatech) was used to measure the absorbance of each well at 570 nm and IC_50_ values were calculated using the probit model. The inhibition rate of cell proliferation was calculated as follows: inhibition rate (%) = 1 − *A*_570_ (test)/*A*_570_ (control) × 100%. Data were calculated from three independent experiments, each performed in triplicate.

### 3.4. Giemsa Staining

A549 cells were treated by bufalin as describe above and the cells were collected and put on the slides. The slides were then rinsed with sterile water and flooded with freshly prepared Giemsa’s stain solution (BDH Chemical Ltd) for 5 min. After three times of rinsing in sterile water, the cells were examined for morphological changes using a microscopy (Nikon, TMS) with 200× magnification.

### 3.5. Flow Cytometry Analysis

A549 cells were treated by bufalin as describe above and the cells were collected for propidium iodide (PI) staining. Briefly, the cells were fixed in 70% ethyl alcohol at 4 °C overnight, then washed with PBS and incubated with RNAse (10 μg/mL) at 37 °C for 30 min. Next the cells were incubated with PI (final concentration 10 μg/mL) for 30 min in the dark. Samples were analyzed using a FACSCalibur flow cytometer within 30 min after the staining.

### 3.6. Western Blot Analysis

A549 cells were treated by bufalin as describe above and the cells were collected for Western blot analysis. Briefly, the cells were lysed in RIPA buffer for 40 min on the ice. Lysates were collected after centrifuging at 12,000 rpm for 20 min at 4 °C. Protein levels were quantified using Lowry method. Equivalent amounts of protein (50 μg/lane) were separated by 15% sodium dodecyl sulfate-polyacrylamide gel electrophoresis (SDS-PAGE) and transferred to PVDF membranes. The membranes were blocked in PBS containing 5% non-fat dry milk (w/v) for 1 h, and then incubated with primary antibodies overnight at 4°C. The membranes were then incubated with HRP-conjugated secondary antibodies at room temperature for 30 min, and developed using enhanced chemiluminescence reagent and exposed to X-ray film.

### 3.7. Statistical Analysis

The data were expressed as the mean ± standard deviation (SD) from experiments performed in triplicate. T test was used to identify statistically significant differences between the experimental and control groups. The statistical analyses were performed using the SPSS software 13.0 (SPSS Inc., Chicago, IL, USA). A value < 0.05 was considered statistical significance.

## 4. Conclusions

Apoptosis is a cell defense mechanism to eliminate malignant cells and plays an important role in preventing tumor development. In fact, many anti-cancer drugs function primarily to induce apoptosis through regulating apoptosis-associated signaling [13,14]. Bcl-2 family members are known to regulate the mitochondrial apoptosis pathway. Anti-apoptotic protein Bcl-2 is located in the mitochondrial outer membrane where it inhibits pro-apoptotic molecule Bax to maintain the integrity of the mitochondrial outer membrane and prevent the release of Cyt-c and other apoptotic factors, thereby protecting cells from apoptosis [15].

In this study we demonstrate that bufalin treated A549 lung cancer cells exhibited significantly higher rates of apoptosis than control treated cells. These results were consistent with the MTT assay showing that bufalin inhibited the proliferation of A549 cells in a dose and time dependent manner. Therefore, we conclude that the loss of viability of A549 cells treated by bufalin is partly due to the induction of apoptotic cell death.

Notably, we found that bufalin induced apoptosis is associated with the downregulation of Bcl-2 expression and upregulation of Bax expression in A549 cells, consistent with previous report that overexpression of Bcl-2 protein inhibited bufalin-induced apoptosis of leukemia cells [16]. In addition, by Giemsa staining we observed cytoplasmic shrinkage, nuclear condensation and the formation of apoptotic bodies in A549 cells treated with bufalin. These are characteristic apoptotic morphology, thus confirming that bufalin induces the apoptosis of lung cancer cells. Furthermore, our results show that the application of caspase-3 inhibitor Z-DEVD-fmk could significantly block the apoptosis of A549 cells treated with bufalin, indicating that bufalin-induced apoptosis involves the activation of caspase-3.

Livin is a newly discovered member of inhibitor of apoptosis protein family [17]. The BIR domain of livin has a new type of zinc finger structure which could bind Caspase-3 to inhibit its activity, thereby inhibiting apoptosis [18]. Interestingly, in this study we found that with the duration of bufalin treatment, livin expression was significantly inhibited in A549 cells, accompanied by increased activation of Caspase-3 and increased apoptosis. A recent study showed that inhibiting livin could induce the apoptosis of human bladder cancer cells via a mechanism involving caspase 3 [19]. Based on these data we speculate that bufalin downregulates the expression of livin. Consequently, Caspase-3 gets activated due to the lack of inhibition by livin and apoptosis ensues.

PI3K/Akt pathway plays critical roles in mammalian cell survival and resistance to apoptosis [20]. PI3K/Akt signaling has been shown to be activated in a variety of cancers and activated Akt acts to phosphorylate Bad and Caspase-9 or activate NF-κB pathway to promote the resistance of cancer cells to apoptosis [21–24]. However, the functional role of PI3K/Akt pathway in bufalin induced cancer cell apoptosis remains largely unclear. In the present study we found that bufalin treatment reduced the level of phosphorylated Akt when compared to control cells, indicating the inhibition of Akt activation. Furthermore, by employing Akt specific inhibitor LY294002 we could demonstrate the synergistic effects of bufalin and LY294002 on the induction of apoptosis and regulation of apoptosis related proteins in A549 cells. The expression of anti-apoptotic molecules such as Bcl-2 and livin was substantially downregulated by pretreatment with LY294002 followed by bufalin treatment of A549 cells, while the expression of pro-apoptotic protein Bax and the activation of Caspase-3 were substantially upregulated. Collectively, these results suggest that bufalin inhibits the activation of PI3K/Akt pathway to promote the apoptosis of cancer cells. However, further studies are needed to elucidate the mechanism by which bufalin modulates the activation of PI3K/Akt and other signaling pathways that are crucial for cancer cell survival and chemoresistance.

In conclusion, in the present study we demonstrate that bufalin inhibits the proliferation of A549 cells by inducing the apoptosis in a dose and time dependent manner. Mechanistically, we found that bufalin inhibits the activation of PI3K/Akt pathway to regulate the expression of pro-apoptotic and anti-apoptotic proteins and promote the activation of Caspase-3, leading to the execution of apoptosis. Interestingly, the marked ability of bufalin to synergize with Akt inhibitor to induce cancer cell apoptosis suggests that bufalin is a potential regimen for combined chemotherapy to overcome the resistance of cancer cells to chemotherapeutics induced apoptosis.
